# Highly Stable Zr(IV)-Based Porphyrinic Metal−Organic Frameworks as an Adsorbent for the Effective Removal of Gatifloxacin from Aqueous Solution

**DOI:** 10.3390/molecules23040937

**Published:** 2018-04-18

**Authors:** Jing-Jing Chen, Li-Juan Wang, Gui-Ju Xu, Xia Wang, Ru-Song Zhao

**Affiliations:** Key Laboratory for Applied Technology of Sophisticated Analytical Instruments of Shandong Province, Shandong Analysis and Test Center, Qilu University of Technology (Shandong Academy of Sciences), Jinan 250014, China; ffcjj163@163.com (J.-J.C.); wape0203@126.com (L.-J.W.); xuguiju2013@126.com (G.-J.X.); zhaors1976@126.com (R.-S.Z.)

**Keywords:** Zr(IV)-based porphyrinic metal−organic frameworks, gatifloxacin, removal

## Abstract

Water stable Zr-metal–organic framework nanoparticles (PCN-224 NPs, PCN refers to porous coordination network) have been solvothermally synthesized. PCN-224 NPs show spherical shape with smooth surface and particle size of approximately 200 nm. PCN-224 NPs can be stable in acid and aqueous solutions, as confirmed by powder X-ray diffraction. Gatifloxacin (GTF) adsorption measurements showed that PCN-224 NPs exhibit a high adsorption capacity of 876 mg·g^−1^. Meanwhile, the adsorption factors, adsorption characteristics, and mechanisms of GTF were investigated in batch adsorption experiments.

## 1. Introduction

Antibiotics are produced by micro-organisms and higher animals and plants throughout their life cycle. Antibiotics can resist pathogens or other types of secondary metabolites, can kill bacteria, and exert good inhibitory and killing effects on mold, mycoplasma, chlamydia, and other pathogenic microorganisms. Therefore, antibiotics are widely used in human medicine, animal medicine, biological science research, agriculture, animal husbandry, and the food industry. However, with the rapid development of human technology, the excessive use of antibiotics has become increasingly serious, and the amount of antibiotics in the ecological environment, especially in the water environment, is continuously increasing [1].

Numerous studies have reported the presence of various antibiotics in waste water [2,3], groundwater [4,5], and surface water [6]. In addition, considerable antibiotic levels have been detected in water treatment plants and drinking water sources around the world [7]. In addition, research shows that the overuse of different types of antibiotics can cause varying degrees of damage to the human body. The indiscriminate use of antibiotics may damage the kidneys, cause gastrointestinal reactions, and even lead to aplastic anemia. Brazilian health authorities believe that the abuse of antibiotics may lead to the spread of bacteria [8]. Furthermore, excessive use of antibiotics by children can damage the development of bodily organs and cause damage to the body’s normal flora, leading to superinfections [9]. The presence of antibiotic residues in aquatic environments continues to threaten human and ecological health, enhance antibiotic-resistant bacteria, and break the ecological balance [10,11,12]. Therefore, effective measures to remove antibiotics from the water environment are crucial.

Several methods, including physical [13], biological [14], degradation [15], membrane filtration [16], ion exchange [17], oxidation [18], semiconductor photocatalysis [19], and adsorption methods [20], have been recently developed to eliminate antibiotics from water environment samples. Adsorption-based methods are portable, reliable, and inexpensive methods for the detection and removal of antibiotic pollutants. The advantages of adsorption-based methods include easy operation, energy saving, and high efficiency. Despite certain progress in this area, considerable efforts are still needed. The challenge in developing adsorption methods lies in the selection of adsorbents. In recent years, numerous high-performance adsorbents have been developed with continuing research on the treatment of antibiotic pollution. These adsorbents include carbonaceous [21], mineral [22], resin [23], mesoporous material [24], molecular imprinted polymer [25], and metal adsorbents [26]. However, due to the difficulty in modifying pores, the application of conventional porous materials, such as activated carbon, zeolites, and aluminosilicates, is limited to a certain extent.

As a new type of porous materials, metal–organic frameworks (MOFs) have attracted increasing attention because of their permanent porosity, high surface area, and the tunable chemical and physical properties of their pores. These properties make MOFs suitable for a variety of applications, in particular for various applications related to adsorption, such as solid phase microextraction [27], solid phase extraction [28], and pollutant adsorption [29]. Therefore, using MOFs to remove organic pollutants is feasible.

In MOFs, Zr-MOFs nanoparticles (NPs) have attracted increasing attention due to their specific electronic and optical properties, permanent porosity, high surface area, and easily tailorable structures and functions. Zr-MOFs can be used as adsorbents in the removal of nitrofurazone and nitrofurantoin from water [30]. However, few reports are available on the removal of antibiotics by using Zr-MOFs NPs as adsorbents.

**Figure 1:**
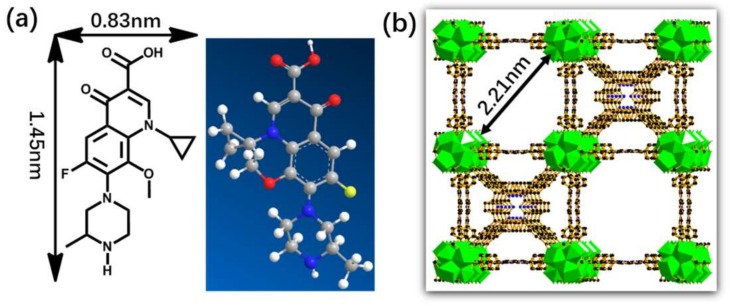
(**a**) Chemical structure of gatifloxacin; (**b**) Crystal structure of PCN-224.

In this paper, Zr-MOFs (PCN-224, PCN denotes porous coordination network) NPs were synthesized [31] and used for the adsorption of gatifloxacin (GTF) in aqueous solution (Figure 1). Meanwhile, the adsorption factors and characteristics of the representative fluoroquinolone antibiotic, GTF, were studied in batch adsorption experiments.

## 2. Experimental

### 2.1. Materials and Instruments

GTF was obtained from Sigma Aldrich Chemical Co. (99% purity, Burlington, NJ, USA). Methyl 4-formylbenzoate, *N,N*-dimethylformamide (DMF), acetone, pyrrole, propionic acid, benzoic acid, tetrahydrofuran, CH_3_OH, KOH, and ZrOCl_2_·8H_2_O were obtained from commercial sources (Alfa Aesar (Tewksbury, MA, USA), Energy Chemical (Shanghai, China), TCI (Shanghai, China), and J & K Scientific (Beijing, China) and were used without further purification unless otherwise mentioned. Tetrakis(4-carboxyphenyl)porphyrin (H_2_TCPP) ligand was synthesized according to the literature [31].

^1^H-NMR spectra were recorded on a 400 MHz Varian INOVA spectrometer (Unity INOVA 400, Varian, Palo Alto, CA, USA) and referenced to the residual solvent peak. Powder X-ray diffraction measurements were carried out on an analytical X-Pert pro diffractometer (Rigaku, Tokyo, Japan) with Cu-Kα radiation (γ = 1.5478 Å). Thermogravimetric analysis (TGA) was performed on a Mettler Toledo TGA (Zurich, Switzerland) under N_2_ flow and heated from room temperature to 900 °C at 10 °C/min. Elemental analyses (C, H, and N) were obtained on a PerkinElmer 240 elemental analyzer (EA 2400 II, Waltham, MA, USA). Infrared (IR) spectroscopy spectra were collected on a Nicolet 330 FTIR Spectrometer (Thermo Fisher Scientific, Waltham, MA, USA) within the 4000–400 cm^−1^ region. Morphology was obtained using scanning electron microscopy (SEM) (S-4800, Hitachi, Marunouchi, Japan, 3.0 kV). In addition, the concentrations of GTF were measured using a UV–Vis spectrophotometer (UV, Shimadzu, Kyoto, Japan, 190–900 nm). Gas adsorption–desorption measurements of N_2_ (99.995%) on PCN-224 NPs was collected on the Micromeritics ASAP 2020 surface area and pore size analyzer (Norcross, GA, USA). The as-synthesized crystals of PCN-224 were exchanged twice with dry acetone. The acetone-exchanged sample was degassed at 393 K for 12 h until the exhaust gas rate was 5 mm Hg·min^−1^ to produce the activated phases of PCN-224 for gas sorption measurements.

### 2.2. PCN-224 NP Synthesis

A total of 150 mg ZrOCl_2_·8H_2_O and 1500 mg of benzoic acid were ultrasonically dissolved in 50 mL of DMF in a round-bottomed flask. Thereafter, 50 mg H_2_TCPP was added into the system, and the solution changed from colorless to dark purple. The mixture was heated in 120 °C for 1 h. After being cooling down to room temperature, dark brown powders were collected by centrifugation and washed for thrice with DMF and acetone (35 mg, 46% yield). FT-IR (KBr, cm^−1^): 3440(m), 2958(w), 1708(w), 1612(s), 1545(s), 1411(s), 1314(s), 1190(m), 1015(s), 866(w), 816(w), 779(m), 722(s); The analytical calculated elemental contents (%) for PCN-224 NPs are C, 44.04%; H, 2.62%; and N, 4.28%, while the founded results are: C, 45.49%; H, 3.02%; N, 4.07%.

### 2.3. Adsorption and Removal of GTF by PCN-224 NPs

An 800 mg·L^−1^ standard stock solution of GTF was prepared by dissolving 0.4 g antibiotic in distilled water and diluting to a 500 mL brown volumetric flask. The standard stock solutions will be diluted to the required concentration in the experiment and stored in the dark below 4 °C.

A total of 10 mL of 300 mg·L^−1^ GTF solution and 2 mg PCN-224 powder were introduced into 20 mL brown vials and mixed thoroughly by an ultrasonic cleaner for several seconds. Then, the vial was placed in a water bath oscillator with a shaking speed of 280 rpm at 25 °C for adsorption. After shaking for 240 min, the adsorption equilibrium was reached, and the suspension was centrifuged using a 0.22 μm. Then, the filtrate was collected, diluted, and analyzed immediately by UV–Vis spectrophotometry at 287 nm. Adsorption experiments were carried out under the same conditions. To evaluate the influence of pH, we adjusted the pH of GTF solutions from 3 to 9.5 by using 0.02 mol·L^−1^ NaOH and HCl. When we studied the effect of ionic strength, different amounts of NaCl particles were dissolved in the GTF solution, and the ionic strength were 0–2.5 mol·L^−1^. To study the effect of contact time, the contact time was in the range of 5–900 min. Adsorption isotherm and thermodynamic studies were carried out with initial GTF concentrations of 100–400 mg·L^−1^ at controlled temperature levels of 298, 308, and 318 K.

## 3. Results and Discussion

### 3.1. Characterization of PCN-224 NPs

**Figure 2:**
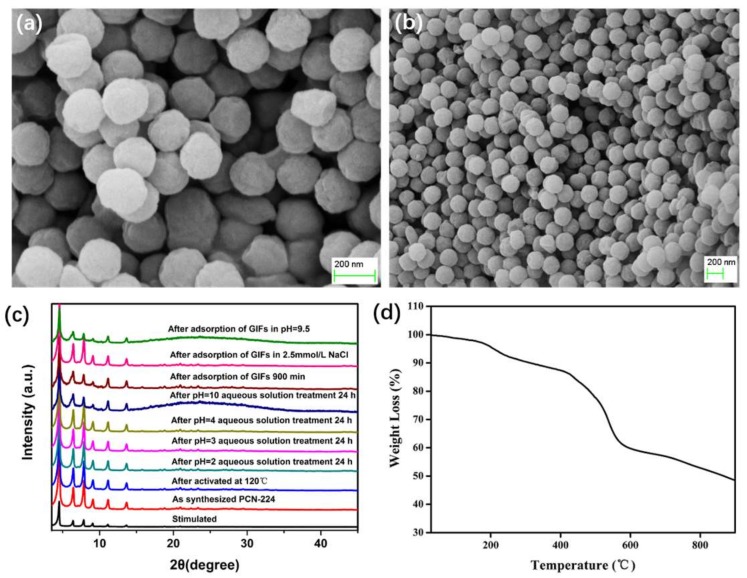
(**a**,**b**) SEM; (**c**) XRD; and (**d**) TGA images of PCN-224 NPs.

The crystallographic structure and phase purity of PCN-224 NPs were examined by powder X-ray diffraction (Figure 2c). No additional peaks from impurities were detected compared with simulated XRD from the crystal structure of PCN-224, indicating high purity of the product. SEM results for the as-obtained PCN-224 NPs showed that the morphology of MOFs was spherical in shape with macroscopic and microscopic images. Moreover, solid PCN-224 microcubes were observed with smooth surface and size of ~200 nm (Figure 2a,b). To examine the thermal stability of PCN-224 NPs, we investigated the samples by using TGA. As shown in Figure 2d, the TGA curves indicate that PCN-224 NPs are thermally stable up to 430 °C.

**Figure 3:**
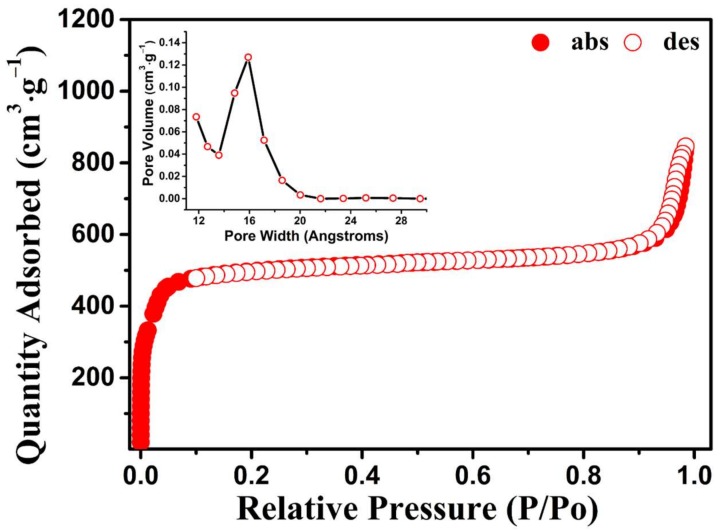
N_2_ adsorption–desorption isotherm and pore size distribution of PCN-224 NPs.

Establishment of permanent porosities is an important goal in MOF research. As shown in Figure 2c, the active phases are highly crystalline and remained almost the same as its as-synthesized phase. The N_2_ gas sorption curves of PCN-224 NPs at 77 K were recorded to check for porosity (Figure 3). PCN-224 NPs showed type I N_2_ adsorption isotherms at 77 K, suggesting permanent porosity. PCN-224 NPs showed N_2_ gas uptake of 845 cm^3^·g^−1^ at 77 K and 1 bar. The Brunauer–Emmett–Teller and Langmuir surface areas calculated from the N_2_ sorption isotherm were 1900 and 2234 m^2^·g^−1^, respectively. The pore volume of PCN-224 NPs was calculated as 0.95 cm^3^·g^−1^. Pore size distributions were determined with NLDFT calculations from N_2_ adsorption isotherms at 77 K, corresponding to the PCN-224 NP pore size of 15.9 Å.

### 3.2. Adsorption Capacity and Removal Percentage

The performance of adsorbent is usually depicted by adsorption capacity and removal percentage. The adsorption capacity, *q*_e_ (mg·g^−1^), *q*_t_ (mg·g^−1^), and the removal percentage was calculated using Equations (1) to (3), as follows:

(1) $$q_{t}=\frac{V\left(C_{0}-C_{t}\right)}{m}$$

(2) $$q_{e}=\frac{V\left(C_{0}-C_{e}\right)}{m}$$

(3) $$\mathrm{Removal}\;\mathrm{percentage}\;\left(\%\right)=\frac{C_{0}-C_{e}}{C_{0}}\times 100$$

where *C*_0_ is the initial GTF concentration (mg·L^−1^), *C*_e_ is the residual GTF concentration at equilibrium (mg·L^−1^), *V* is the volume of solution (L), and *m* is the mass of dry PCN-224 NPs (g).

In the pre-experiment, PCN-224 NPs showed excellent adsorption performance for GTF. All GTF (100%) was removed within 15 min when 10 mg PCN-224 powder was dispersed in 10 mL (80 mg·L^−1^) of GTF solution. This result indicated that the prepared PCN-224 NPs may offer excellent adsorption potency for the removal of GTF from aqueous solutions.

### 3.3. Effect of Contact Time

**Figure 4:**
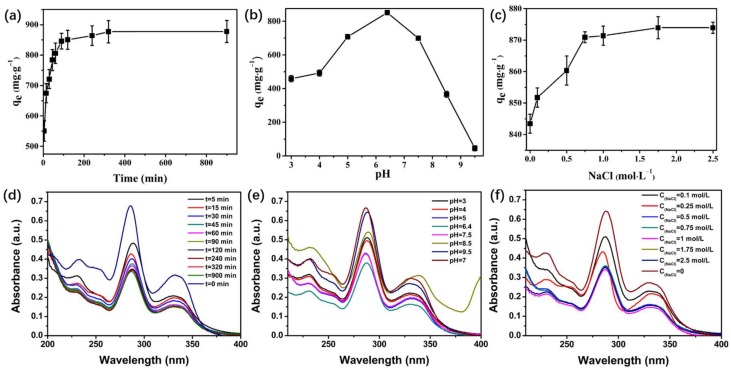
(**a**,**d**) Effect of contact time on the adsorption capacity of GTF; (**b**,**e**) Effect of pH on the adsorption capacity of GTF; (**c**,**f**) Effect of ionic strength on the adsorption capacity of GTF.

In the adsorption process, contact time is an important factor affecting adsorption capacity. The amount of antibiotics adsorbed at different exposure times is shown in Figure 4a. As shown in the figure, the adsorption capacity is rapidly adsorbed in aqueous solution, and then the adsorption capacity increases slowly after the adsorption capacity reaches the maximum adsorption capacity. Therefore, the optimal adsorption time was 240 min. This substantial adsorption amount indicates that molecules can gradually diffuse into the interior of the adsorbent.

### 3.4. Effect of pH

The effect of solution pH on the adsorption of GTF onto PCN-224 NPs was investigated in the pH range of 3–9.5, and the results are shown in Figure 4c. As shown in the figure, GTF adsorption onto PCN-224 NPs is highly dependent on solution pH. The optimal pH for adsorption was in the pH range of 5–7.5, with an average adsorption capacity of 600 mg·g^−1^. Thus, PCN-224 NPs can effectively remove GTF because of the electrostatic attraction and repulsion between GTF and the surfaces of the adsorbent under low pH conditions. When the pH exceeds 8, the structure of PCN-224 is unstable, resulting in a significant decrease in the adsorption capacity.

### 3.5. Effect of Ionic Strength

To study the effect of ionic strength on the adsorption of GTF onto PCN-224 NPs, we added different amounts of NaCl onto the solution. The results are shown in Figure 4e. The adsorption capacity of GTF decreased with increasing ionic strength. For NaCl, the adsorption capacity increased significantly with the incremental NaCl, increasing from 0 mol·L^−1^ to 0.75 mol·L^−1^. This finding is attributed to the dissolution of GTF in aqueous solution being gradually restrained by increasing NaCl concentration. The solubility of GTF decreases due to the salting out effect, thereby impelling the diffusion of GTF to the hydrophobic surface of PCN-224 NPs and increasing the adsorption capacity.

Moreover, the adsorption capacity showed no marked change with further increase in NaCl concentration to 2.5 mol·L^−1^. As shown in Figure 4e, the variation in adsorption capacity stops after the NaCl in the solution is approximately 1.75 mol·L^−1^. This result suggested that electrostatic competition occurs and plays a certain role during adsorption.

### 3.6. Adsorption Kinetics

**Figure 5:**
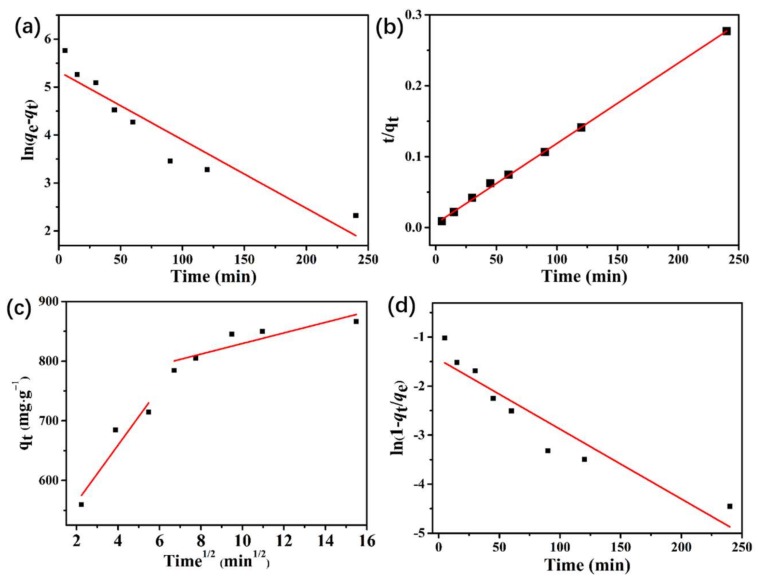
Kinetic models for GTF adsorption on PCN-224 NPs; (**a**) pseudo-first-order; (**b**) pseudo-second-order; (**c**) intra-particle diffusion; and (**d**) liquid film diffusion models.

To study the relationship between the structure of adsorbent and adsorption performance, we used the data in the adsorption equilibrium time experiment to fit the kinetic model. Pseudo-first-order, pseudo-second-order, intraparticle diffusion kinetic, and liquid film diffusion models were used to describe the adsorption process. The four linear forms can be expressed as follows:

(4) $$\mathrm{Pseudo}\text{-}\mathrm{first}\text{-}\mathrm{order}:\;\ln(q_{e}-q_{t})=\ln\left(q_{e}\right)-k_{1}t$$

(5) $$\mathrm{Pseudo}\text{-}\mathrm{second}\text{-}\mathrm{order}:\;\frac{t}{q_{t}}=\frac{1}{k_{2}q_{e}^{2}}+\frac{t}{q_{e}}$$

(6) $$\mathrm{Intraparticle}\;\mathrm{diffusion}\;\mathrm{kinetic}\;\mathrm{model}:\;q_{t}=k_{d}t^{1/2}+I$$

(7) $$\mathrm{Liquid}\;\mathrm{film}\;\mathrm{diffusion}\;\mathrm{model}:\;\ln\left(1-\frac{q_{t}}{q_{e}}\right)=-k_{f}t+A$$

where *q*_e_ (mg·g^−1^) and *q*_t_ (mg·g^−1^) are the amounts of GTF absorbed on the sorbent in equilibrium and at time *t* (min), respectively. *k*_1_ (min^−1^) and *k*_2_ (g·mg^−1^·min^−1^) are the rate constants of the first-order and second-order adsorptions, respectively, and *k*_d_ (g·mg^−1^·min^−1/2^) is the rate constant of intraparticle diffusion. I is a parameter related to the thickness of the boundary layer: increasingly high I means that large boundary layer effect. *k*_f_(*h* − 1) is the rate constant of liquid film diffusion, and A is the liquid film diffusion constant. Figure 5 shows the fitting results by four kinetic models, the corresponding kinetic parameters are listed in Table 1. The corresponding experimental data fitting of the pseudo-second-order model is better than those of the other kinetic models. Meanwhile, the calculated adsorption capacity (*q*_e_,_cal_) of the pseudo-second-order model of 877.1929 mg·g^−1^ closely approximates the experimental value (*q*_e_,_exp_), that is, 876.7694 mg·g^−1^. These results indicate that chemical sorption is relatively dominant and controls the adsorption. The piece-wise linear regressions observed in the intraparticle diffusion plot (Figure 5c) indicate that two distinct regions are included in the adsorption corresponding to two different factors, namely, external mass transfer and intraparticle diffusion. The first sharp linear region is a diffusion adsorption stage, which is attributed to the diffusion of GTF molecules through the solution to the adsorbent’s external surface. The second linear region is a gradual adsorption stage, at which the intraparticle diffusion is rate controlled (intraparticle diffusion). The liquid film diffusion model (Figure 5d) yields a relatively low correlation coefficient (*R*^2^) value to fit the experimental data, proving that the rate-limiting step is mainly the molecular diffusion of GTF in the PCN-224 NPs.

### 3.7. GTF Adsorption Isotherms

The equilibrium isotherms at various temperature levels were studied by varying the initial concentration of GTF. Two well-known isotherm models, namely the Langmuir and Freundlich models, were employed in this study to analyze the experimental data.

The equation linear form of the Langmuir model is as follows:

(8) $$\frac{1}{q_{e}}=\frac{1}{k_{2}q_{e}^{2}}\times \frac{1}{C_{e}}+\frac{1}{q_{m}}$$

The equation linear form of the Freundlich model is as follows:

(9) $$\ln\left(q_{e}\right)=\ln\left(K_{F}\right)+\frac{1}{n}\ln\left(C_{e}\right)$$

where *q*_m_ is the Langmuir monolayer adsorption capacity (mg·g^−1^), *q*_e_ is the amount of GTF adsorption at equilibrium (mg·g^−1^), *C*_e_ is the liquid-phase concentration of GTF (mg·L^−1^) at equilibrium, and b is the Langmuir constant (L·g^−1^) related to adsorption energy. *K*_F_ is the Freundlich constant (mg·g^−1^ (mg·L^−1^)^−1/*n*^), and 1/*n* is the heterogeneity factor describing the adsorption intensity.

**Figure 6:**
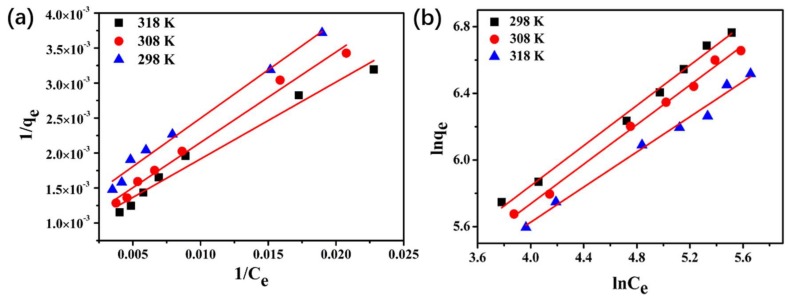
Equilibrium adsorption isotherms fitted by the (**a**) Langmuir and (**b**) Freundlich models.

Figure 6 shows the isotherm of GTF and the fitting results by Langmuir and Freundlich models. The corresponding isothermal parameters and correlation coefficients are presented in Table 2. According to the *R*^2^ values, the Freundlich model is more suitable than the Langmuir model for describing adsorption. The maximum adsorption capacity (*q*_m_) was decreased with increasing temperature, indicating that lower temperature facilitates GTF adsorption onto PCN-224 NPs and that the adsorption is an exothermic process. The 1/*n* values for the three temperatures studied were in the range of 0–1, which illustrated a favorable adsorption process.

### 3.8. GTF Adsorption Thermodynamics

The thermodynamic parameters reflect the feasibility and spontaneous nature of the adsorption. The thermodynamic feasibility for GTF adsorption onto PCN-224 NPs has been demonstrated by evaluating the thermodynamic parameters, including entropy (∆*S*^θ^), enthalpy (∆*H*^θ^), and the Gibbs free energy (∆*G*^θ^), which are calculated using the following equations:

(10) $$K_{c}=\frac{q_{e}}{C_{e}}$$

(11) $$∆G^{\theta }=-RT\ln K_{c}$$

(12) $$\ln K_{c}=\frac{∆S^{\theta }}{R}-\frac{∆H^{\theta }}{\mathrm{RT}}$$

where *q*_e_ is the amount of antibiotic absorbed on the sorbent at equilibrium, *C*_e_ is the solution concentration at equilibrium, *K*_c_ is the distribution coefficient, R is the molar gas constant (8.314 J·mol^−1^·K^−1^), and *T* is the temperature (K). The values of ∆*S*^θ^ and ∆*H*^θ^ can be calculated from the intercept and the slope of the linear plots shown in Figure 6. Moreover, the thermodynamic parameters are summarized in Table 3. The ∆*H*^θ^ value was −16.609 KJ·mol^−1^, implying that adsorption is an exothermic reaction and that lower temperature is beneficial for absorbing GTF. The ∆*G*^θ^ and ∆*S*^θ^ values were negative, indicating that the adsorption of GTF onto PCN-224 NPs may be a spontaneous, physical sorption process.

## 4. Conclusions

In summary, Zr-MOF PCN-224 NPs were prepared and their adsorption behaviors were characterized and studied. Good removal capability for GTF from aqueous solution was obtained by the prepared PCN-224 NPs. The effect of contact time, pH, and ionic strength were considered and optimized. The adsorption kinetics, thermodynamics, and isotherms of GTF were calculated to characterize the adsorption behavior of GTF on PCN-224 NPs. The results exhibited high GTF adsorption and removal rates of PCN-224 NPs, and the pseudo-second-order model for adsorption kinetics was an excellent fit (*R*^2^ = 0.999). The adsorption isotherm was more consistent with the Freundlich model than the Langmuir model, indicating that the adsorption of GTF on PCN-224 NPs involves multilayer adsorption. The adsorption capacity of GTF on PCN-224 NPs was negatively related to the temperature. Moreover, the adsorption process was spontaneous and exothermic.

## Figures and Tables

**Table 1:** Kinetic parameters for the adsorption of GTF onto PCN-224.

***q*_e,exp_ (mg·g^−1^)**	**Pseudo-First Order**			**Pseudo-Second Order**		
	***q*_e,cal_ (mg·g^−1^)**	***k*_1_ × 10^−3^ (min^−1^)**	***R*^2^**	***q*_e,cal_ (mg·g^−1^)**	***k*_2_×10^−3^ (g·mg^−1^·min^−1^)**	***R*^2^**
876.7694	205.0779	14.24	0.8741	877.1929	2.3	0.9997
**Intraparticle Diffusion**			**Liquid-Film Diffusion**			
***k*_d_ (mg·g^−1^·min^−1^)**		**I**	***R*^2^**	***k*_f_ (min^−1^)**	**A**	***R*^2^**
8.8577		740.97517	0.7251	0.01424	−1.45284	0.8741

**Table 2:** Adsorption isotherm models parameters.

Isotherm Model	Parameters	Temperature (K)		
		298	308	318
Langmuir	b	8.106 × 10^−3^	7.744 × 10^−3^	6.654 × 10^−3^
	*q*_m_	1223.135	1163.643	892.857
	*R*^2^	0.973	0.986	0.983
Freundlich	*K*_F_	33.693	30.905	28.817
	*1/n*	0.527	0.594	0.603
	*R*^2^	0.992	0.994	0.985

**Table 3:** Adsorption thermodynamic parameters.

Temperature (K)	ln*K*_c_	∆*G*^θ^ (KJ·mol^−1^)	∆*H*^θ^ (KJ·mol^−1^)	∆*S*^θ^ (KJ·mol^−1^)
298	2.3619	−5.85	−12.61	0.0228
308	2.1347	−5.46		
318	2.0434	−5.40		
